# Manuscript Writing and Publication Workshop: An Invoking Pilot Study on Enhancing Cognitive Research Capabilities in Health Sciences Institutes of Pakistan

**DOI:** 10.7759/cureus.8802

**Published:** 2020-06-24

**Authors:** Mehwish Hussain, Rehana Rehman, Mukhtiar Baig

**Affiliations:** 1 Biostatistics, Imam Abdurrahman Bin Faisal University, Dammam, SAU; 2 Biological and Biomedical Sciences, Aga Khan University, Karachi, PAK; 3 Clinical Biochemistry, King Abdulaziz University, Jeddah, SAU

**Keywords:** interactive workshop, research manuscript, manuscript writing, publications, cognitive abilities, capacity building, pakistan

## Abstract

Background

With an upsurge in research in developing countries, researchers from allied sciences need to augment their skills for disseminating research work worldwide. Training workshop is one of the quick interventions which can enhance writing skills and ease research publication.

Objective

We designed this research to explore the perception of the faculty of different higher education institutes (HEIs) regarding manuscript writing and to assess the impact of these workshops in the improvement of cognitive capabilities of preliminary researchers in Pakistan.

Methodology

We conducted workshops in HEIs of Sindh, Pakistan. Contents of the workshop covered algorithm of writing manuscript and related descriptions, choice of quality journals, correspondence with the editor, and dealing with rejection. The knowledge of the participants was assessed by 15 items pre and post evaluation tests. McNamar's test assessed the significance of the change in knowledge. Kruskal Wallis test was performed to check the difference in the opinion of workshop quality among different institutes.

Results

A significant improvement was observed in participants’ knowledge on the readers’ prospects (P=0.001), the algorithm of writing (P<0.001), interpretations of results (P<0.001), and selection quality journal for publication (P <0.001). The agreement with the workshop's quality based on need, knowledge, and content was above average.

Conclusion

The participants’ response regarding the effectiveness of manuscript writing for publication workshop was overwhelmingly positive, and there was a significant impact on the knowledge of the participants. There is a need for research training that will help for better capacity building in different HEIs of Pakistan.

## Introduction

The progress of any country is gauged by the quality of research papers published from that country. Now science not only flourishes in the scientific laboratories but also the educational institutes. In developing countries, there is a need to adopt a research-oriented approach by higher education institutes (HEIs). A research-oriented institute would be able to inculcate research skills among its students.

In Pakistan, there is a mushroom growth of degree awarding institutes in the private sector [1]. Still, these neither allocate sufficient budget for research funding nor provide training facilities to their faculty for research writing. Even the medical colleges’ condition is the worst because the research does not come on their priority list, and there is no emphasis from Pakistan Medical and Dental Council (PMDC) as well. Although, almost a decade before, the Higher Education Commission (HEC) of Pakistan imposed policies in HEIs to augment research culture [2,3]. Nevertheless, projection in research publications from all sectors of medical institutes is slow or having low quality in terms of good scientific publications in impact factor journals as compared to other countries [3,4]. We have one good example from a private sector institution that has research on their priority list and provide sufficient funding and training to their faculty and students and also motivates the students and faculty towards research [5].

All over the world, the developing countries are in the surge of inducing scientific research and its dissemination. Scientific writing demands keen interest, learning, and practices to disseminate research work. Nevertheless, health sciences researchers find impedance in publishing scientific work [6-9]. Medical professionals’ limited reading and writing practices hurdle good scientific journals [10] and cause an under-representation of health status from developing countries [11]. The literature search strategy enhances the capability to learn quality research work and to become competent researchers and writers [7,12]. On the other hand, institutional support is the catalyst to produce good publications in the field of health sciences [13]. However, preliminary researchers hinder without a productive learning environment and expert instructors’ cognitive learning [8]. Consequently, many health hazard conditions are not enunciated worldwide, especially from developing countries [10].

Scientific writing is essential for researchers, but due to a lack of skills and interests, many good research pieces do not place their positions in quality journals. There is a need for intervention to improve writing skills in preliminary researchers [8]. Since all the researchers cannot attend a full-time research course, the training workshop is one of the quick interventions that can enhance writing skills and ease research publication [11,12]. These interventions partly focus on practical matters like getting started on writing, increasing productivity and satisfaction, and getting published [13]. It is a fact that “without publication, science is dead.”

The reputed journals reject several of the manuscripts because of the poor manuscript writing skills and flaws in the study designs. Then, the researchers are compelled to send their manuscripts to sub-standard and non-indexed journals. By keeping these points in mind, we designed a very interactive workshop for enhancing the manuscript writing skills among the teaching faculty of different HEIs. Over two years, we conducted six five workshops in different HEIs in Pakistan to transfer the best of our knowledge and skills to various HEIs faculty members. We designed this research to explore the knowledge and perception of the faculty of HEIs regarding manuscript writing and to assess these workshops' improvement in the cognitive capabilities of preliminary researchers in Pakistan.

## Materials and methods

Study design and setting

There are 22 medical HEIs affiliated with ten universities in Sindh, as recognized by Pakistan, Medical and Dental College [1]. This mixed-method study was carried out in five different medical institutes. We approached three more medical HEIs for facilitating the workshop but could not get permission from the relevant authority for the same. Five workshops were conducted from November 2015 to September 2016. As a retrospective workshop data analysis, the Institutional Review Board approval was not sought. However, permission for conducting workshops was taken from the relevant authorities. This study was conducted in accordance with the Helsinki Declaration of Human Rights, and the identity of institutes and participants was not disclosed. To preserve confidentiality, we labeled the participated HEIs name as A, B, C, and D. The same workshop in HEI labeled D was conducted twice because of their demands based on the participant's strong recommendations in the first workshop.

Contents covered

The content of the workshop and evaluation questionnaires were then modified after scrutinizing responses of the participants in the pilot workshop. The content of the workshop covered discussion on the introduction of research writing, checklist of writing a different type of papers, the algorithm of writing research, the structure of methodology, pattern of writing results, data presentations, discussion, conclusion, abstract, title of the manuscript, publication ethics, choice of the journal, correspondence with editors, leading reasons and dealing with rejections and considerations in revising and submitting the manuscript. The workshops were kept interactive with the inclusion of different types of activities such as pair-discussion, activity articles, quick group presentation, and facilitator-participant communication environment throughout the workshop.

Instruments for evaluation

Fifteen test items assessed the knowledge of the participants on these features of publications. Each of the items surmised four options with one best answer to be chosen by the participants based on the content of the workshop. The test was distributed to the participant before and after the workshop. Participants were also asked to fill an evaluation form after the workshop, which helped to evaluate workshop facilitation and the need for similar activities in the institute. The response of this form was asked in 5 points Likert scale from strongly disagree to strongly agree. The workshop took an average of five hours in the delivery of the content and discussion. Writing for the reader’s mind increases the likelihood of publication in quality journals in each workshop’s take-home message. Fifteen days after the workshop, the workshop’s strengths and weaknesses were discussed with participants in focus-group discussions and further discussed the implementation of workshop content in their professional life.

Participants

Twenty participants were registered from each institute except in two institutes, where 25 participants were registered for the workshop. The participation rate was varied in each institute. The participants with a rudimentary knowledge of research publications were asked to register for the workshop.

Statistical analyses

The responses in the pretest and posttest of the workshop were classified in binary form as correct or incorrect. McNemar’s test was then performed to assess changes in the responses of the participants. Median with interquartile range, Cronbach’s alpha, and intraclass correlation was reported to express participants’ responses on workshop evaluation. Kruskal Wallis test was performed to assess differences in the response of participants on workshop evaluation across different institutes. The data obtained from the pilot workshop was not incorporated with data obtained from later workshops.

## Results

Participants and participation

A total of 87 individuals participated in the workshops, and the highest number were from HEI C. On the other hand, the highest participation was observed from A in the focus group discussion (Table 1).

**Table 1:** Participants’ status in the workshops at different institutes. *The workshop was conducted twice in the D institute.

Status/Institute	A	B	C	D	D*
Registered	25	20	25	13	7
Participated	22	19	25	13	7
Completed	21	18	16	10	5

Participants’ knowledge

Table 2 presents the knowledge of the participants before and after the workshop. In the pretest, only 13 participants correctly answered the question about the novelty of the research work as the first thing which readers calibrate for a good research paper. While in the posttest, this number augmented to 33 (P=0.005). Recognition of IMRAD (Introduction, Methodology, Results, And Discussion) format for writing a research paper was known to 45 participants only before the workshop. After the workshop, 80 participants correctly answered the question (P<0.001). Sixty-six participants knew that describing “what problem was studied” should be written in the Introduction section of the manuscript. By the end of the workshop, 70 participants correctly replied to this question. Eleven participants could gauge the correct interpretation of the relationship between categorical variables before the workshop. The correct response to this question became twice in the posttest (P=0.03). Less than half (n=41) of the participants knew the correct presentation of tables and figures before the workshop. This proportion rose to 63% as per responses in the posttest (P=0.01). Thirty-five participants incorrectly replied to the content in writing the discussion. A correct response to the same question reached 64.4% of the participants’ answers in the post-test (P<0.001).

Similarly, 61% of the participants did not correctly know about writing the introduction. This proportion decreased to 21% after the workshop (P<0.001). More than half percent of the participants incorrectly answered about settling authorship criteria before the workshop. After the workshop, 56.3% of participants knew that it should be settled before starting the research work. About two-fifth of the participants did not realize the specified quality of a good journal. After the workshop, 80.5% of the participants knew that a high rejection rate and high impact factor defines the good quality of a journal (P<0.001).

**Table 2:** Comparison of participants’ correct knowledge before and after the workshop. N= number of participants, %= percentage in parentheses Q; 1: The first thing which readers look for calibrating research paper as good, is: (Ans. Novel study) Q.2: The general pattern of a research paper is set under the acronym of: (Ans. IMRAD) Q.3: The question “What problem was studied” should be defined in (Ans. Introduction) Q.4: A scanrio showing a positive correlation between smoking and lung cancer. Q.5: While presenting data in tabular format researcher should avoid the presentation of: (Ans. Tables with single variable or only demographic variables) Q.6: A common mistake when writing the discussion section of the manuscript is: (Ans. Repeating statistics which are delineated in results). Q.7: A major consideration when writing the introduction is: (Ans. Description from general to specific aspects of research understudy) Q.8: Authorship criteria should ideally be settled at: (Ans. Planning of the project) Q.9: Relative importance of a journal is assigned by: (Ans. Impact factor) Q.10: All the content of manuscript writing is based on: (Ans. Research question) Q.11: Statement of the conclusion should contain: (Ans. Outcome derived with reference to the objective of the study) Q.12: The title of the study may contain: (Ans. Keyword) Q.13: Consideration which should never to ignore for selecting a target journal: (Ans. We should spend time for reading previous publications of the journal so that we can judge our manuscript aligns with the content required for that journal) Q.14: The validation of quality journal can be inferred with: (Ans. Low acceptance rate and high rejection rate) Q.15: While drafting and submitting the manuscript, the author should not: (Ans. Write manuscript once and submit the draft to the editor)

Questions	Pretest	Post test	P-value
	N (%)	N (%)	
Q1	13 (14.94%)	33 (37.93%)	<0.001
Q2	45 (51.72%)	80 (91.95%)	<0.001
Q3	55 (83.33%)	17 (19.54%)	0.56
Q4	11 (12.64%)	23 (26.44%)	0.03
Q5	41 (47.13%)	55 (63.22%)	0.01
Q6	35 (40.23%)	56 (64.37%)	<0.001
Q7	34 (39.08%)	45 (51.72%)	0.04
Q8	41 (47.13%)	49 (56.32%)	0.15
Q9	53 (60.92%)	70 (80.46%)	<0.001
Q10	40 (45.98%)	43 (49.43%)	0.74
Q11	43 (49.43%)	42 (48.28%)	0.99
Q12	43 (49.43%)	71 (81.61%)	<0.001
Q13	46 (52.87%)	52 (59.77%)	0.31
Q14	21 (24.14%)	50 (57.47%)	<0.001
Q15	17 (19.54%)	23 (26.44%)	0.31

Less than half of the participants knew that all the contents of the manuscript should be based on the research question of the study. The workshop could not significantly augment this knowledge of the participants. The correct statement of conclusion was known to 49% of the participants before the workshop. There was a slight increase in the number of correct responses on this question after the workshop. Less than half of the participants knew that keyword should be included in the title of the study. After the workshop, knowledge of this consideration was increased to 81.6% (P<0.001). Forty-one participants incorrectly replied on considerations of choice of a journal. This consideration was correctly known to nearly 60% of participants in the post-test. The validation of quality journal inferred incorrectly by sixty-six participants before the workshop. The incorrect response to this question decreased to almost half of this number in the workshop post-test. Nearly 80% of the participants did not correctly answer about author’s trait while drafting and submitting the manuscript. There was a 5.4% decreased in this lack of knowledge about the author’s traits after the workshop.

Workshop evaluation

The workshop evaluation forms were returned by 70 (80.4%) participants only. The rest of the 17 forms were missed or did not fill by the participants because they left the workshop after filling the post-test. The workshop was held twice in HEI labeled D, so both groups ' response to the workshop 's efficacy is described in the same column. The intraclass correlation was 0.939, and the value of Cronbach’s alpha was 94.2%, which showed excellent agreement and consistency, respectively, among the responses of participants for workshop evaluation. The median and interquartile range of need for workshop activity was 5 (1), with no significant difference in the responses from different institutes indicating all strongly agreed on average. Participants found timings of the appropriate with an average score of 4 (1). There was a significant difference in the response of the participants for agreeing with the venue and logistic supports (P values = 0.01). Congruency of objectives with the learning needs of participants was strongly agreed by B and D but agreed by A and C (P=0.04). 

The delivery of content for objectives of the workshop was strongly agreed by B while just agreed by the participants from another university (P<0.001). Participants were agreed to enjoy the way contents were delivered with little more variation from D (P=0.02). The response of strong agreement was reported by A and B on the adequacy of interaction level while just agreed was reported by C and D (P<0.001). Agreement on time management was most substantial from B (P<0.001). Participants from the same university also provided strongly agree response for the items, including adequate hands-on experience, quality of handouts, training in the session, was worth the time spent, acquiring desired outcomes, and putting knowledge into practice. The workshop developed better interpersonal skills strongly agreed by B and D, while A and C delineated simple agreement in this regard (P<0.0001) (Table 3).

**Table 3:** Comparison of participants’ responses regarding effectiveness of the workshop. Responses: 1: Strongly disagree, 2: Disagree, 3: Neither agree nor disagree, 4: Agree, 5: Strongly agree *Values are presented as median (interquartile range).

Items*	Overall	A	B	C	D	P Value
There was a need for such activity	5 (1)	5 (1)	5 (1)	5 (1)	5 (1)	0.14
Timing of sessions was appropriate	4 (1)	4 (1)	4 (2)	4 (0)	4 (2)	0.09
The venue was up to the mark	4 (1)	4 (1)	4.5 (1)	4 (0)	5 (1)	0.01
Logistic support was provided	4 (1)	4.5 (1)	5 (1)	4 (1)	5 (1)	0.01
All the sub-events were organized	4 (1)	4 (0)	4 (1)	4 (0)	4 (2)	0.1
Outcome & Objectives were given before the session	4 (1)	4 (1)	5 (0)	4 (1)	4 (1)	<0.001
Objectives were congruent with the learning need of the participant	4 (1)	4 (1)	5 (1)	4 (0)	5 (1)	0.04
Contents delivered as per defined objectives	4 (1)	4 (1)	5 (1)	4 (0)	4 (1)	<0.001
Enjoyed the way contents were delivered	4 (1)	4 (1)	4 (1)	4 (1)	4 (2)	0.02
Focused on teaching-learning environment at this institute	4 (1)	4 (1)	4 (1)	4 (1)	4 (1)	<0.001
Theory & Practice was balanced in these sessions	4 (0)	4 (1)	4 (1)	4 (0)	4 (2)	<0.001
Level of Interaction was adequate	4 (1)	5 (1)	5 (1)	4 (0)	4 (1)	<0.001
Time was managed properly	4 (1)	4 (1)	5 (1)	4 (0)	4 (2)	<0.001
Queries were responded satisfactorily	4 (1)	4 (1)	4 (1)	4 (0)	4 (1)	<0.001
Adequate Hand on experience was provided	4 (1)	4 (1)	5 (1)	4 (0)	4 (0)	<0.001
Handouts were of appropriate quality	4 (1)	5 (1)	5 (0)	4 (0)	4 (1)	<0.001
Training in the sessions was worth the spent time	4 (1)	4 (1)	5 (0)	4 (0)	4 (1)	<0.001
Desired outcomes were acquired	4 (1)	4 (1)	5 (1)	4 (1)	4 (2)	<0.001
Developed better Interpersonal skills in terms of listening, giving comments and receiving criticism	4 (1)	4 (0)	5 (1)	4 (1)	5 (1)	<0.001
Acquired Knowledge can be put into practice	4 (1)	4 (1)	5 (0)	4 (0)	4 (1)	0.01

Responses in the focus group discussion (FGD)

In the FGD, participants strongly appreciated the knowledge gained from the workshops. They got motivation in writing their unfinished manuscript.

“After the workshop, we opened our thesis to write a manuscript from it.”

“Indeed, the workshop did give me some insight as to how I can better my research writing and especially the discussion part.”

Participants also commented on different components of the workshop affirmatively.

“The algorithm of writing manuscript was the best part which will surely help us in easy writing.”

“Checklist made sure that we went through everything, so it was an important accessory.”

“Hands-on activity was a good way to revise every component of the workshop in a short period of time.”

Participants got to know about different checklists of writing guidelines such as IMRAD, Consolidated Standards of Reporting Trials (CONSORT), Strengthening the Reporting of Observational Studies in Epidemiology (STROBE) and Preferred Reporting Items for Systematic Reviews and Meta-Analyses (PRISMA) from this workshop. The existence of any predatory journal, selection of any journal according to the subject of the manuscript, authorship placements, and correspondence with the editor were the important issues that participants lacked knowledge before the workshop.

Few recommendations were shared by the participants to enhance more knowledge during the delivery of the workshop. They asked if separate workshops could be arranged on different topics such as research methodology, reference management, data analysis, and other related. Participants had an issue with the short duration of time of the workshop and the contents covered.

“It should be a whole day workshop and should be conducted step by step; basic for beginners and advanced in the form of series for those who have attended the first workshop.”

## Discussion

In Pakistan, generally, most of the public and particularly private sector HEIs want their faculty to do quality research work and get their papers published in the high impact factor journals. However, these institutes do not provide a conducive environment, required infrastructure, and optimal funding for such activities, and this factor compels the faculty to do substandard research and get that published in non-indexed journals. In this regard, the Government of Pakistan spends enormous resources on overseas research training to flourish a scientific base in the research field [14]. Medical educationists take the number of steps to urge scientific research by journal club presentations and improve the research write-up skills [14]. Nevertheless, the dissemination of research work to the scientific world is comparatively low in healthcare perspectives [15,16]. The little reading and writing practices observed by individuals can be promoted by research culture and training at the medical school level with mentoring [17].

As has been observed by other researchers, participants in our workshop were not positive about the worth of their ideas, had little understanding of writing their thoughts, and were not familiar IMRAD format [18]. They had a flight of research ideas but were not sure whether ideas are worthy of publication. The fears and apprehensions about their writing abilities were similar to another study [13]. Our study elucidated improvement in participants’ knowledge that motivated them to write their research after the workshops. Our study elucidated changes in the participants' awareness, which inspired them to write their research after the workshops.which can be shown by the statement; “After the workshop, we opened our thesis to write manuscript from it.”

Perfection in medical writing skills is likely to improve the submission and publication of research data. Writing is believed to be a starting point, rather than an endpoint of the research process in academic growth and development [18]. In these interactive workshops, we have tried to discuss all these issues and help the participants to think that they can write for scholarly, indexed, and validated journals, irrespective of their position, i.e. from medical students to postgraduates, basic sciences faculty, and clinicians. The group activity gave them a message to share their ideas and experience of writing at a multi-disciplinary forum [19]. The participants in post-workshop evaluation documented medical writing skills were inculcated, and they rated workshops in all aspects, similar to a study by Jawaid et al. [12]. It has been evidenced that similar studies have worked to enhance research culture and mend brain drain of Pakistani researchers [11,12,20]. A study affirmed that workshops on literature search increased the understanding of evidence-based practice among dental researchers [20]. Satisfying response to workshop evaluation indicated the need for similar activities in our academia. The participants in the workshop mentioned that the algorithm for writing manuscripts was the best part that would undoubtedly help them write easily, and the checklist provided ensured that they followed all the steps. They also stated that this workshop gave us an insight into how we could write our research better, particularly the discussion section.

Our study is similar to the study from the Kramer, who observed that enabling the environment facilitates the improvement in writing skills, dissemination of scientific knowledge, publication of research data, and achievement of health care prospects [21]. This study has shown that short, interactive hands-on medical writing workshops are beneficial in improving the participants’ knowledge and skills. The participants appreciated the hands-on activities and said this is an excellent way to review every aspect of the workshop in a brief period. From an institutional perspective, the increased pressure to publish is required for placement as well as promotions in jobs [22]. The overall format of the workshop was designed in such a way that participants could learn from how a topic was perceived to submission to a peer-reviewed journal at the end. The workshop created awareness amongst the participants about the Impact factor, authorship criteria, and caution against predatory journals. The success of such activity is reflected in the suggestion from the participants that a full-day workshop should be held for beginners and series to follow afterward.

We suggest that HEIs should frequently organize such manuscript writing workshops on their campuses. Besides, we recommend that there should be a research methodology module in their educational program, or it could be introduced as an elective. Good manuscript writing skills are as important as good research. A well-written manuscript has bright chances of acceptability in good impact factor journals and publication in a reputable journal. It is not only important for the faculty members but also crucial for improving the institute’s overall ranking. A better ranking attracts bright students and good faculty members and, consequently, enhances the ranking.

Limitations

There are several limitations to our study. Firstly, the sample size is small. Secondly, we have mentioned the initial change in the participant’s knowledge, but we did not evaluate the long-term impact on their attitude and practice that needs a follow-up study for which the present data would help a lot.

## Conclusions

The participants’ response regarding the effectiveness of manuscript writing and publication workshop was overwhelmingly positive, and there was a significant impact on the participants’ knowledge. There is a need for research training that will help for better capacity building in different HEIs of Pakistan. We strongly recommend that there should be a full flash series of workshops on statistics in research, scientific writing, and tackling hurdles in publications.
